# Olfactory–gustatory simultaneity judgments: A preliminary study on the congruency‐dependent temporal window of multisensory binding

**DOI:** 10.1002/brb3.2821

**Published:** 2022-11-30

**Authors:** Naomi Gotow, Tatsu Kobayakawa

**Affiliations:** ^1^ Human Informatics and Interaction Research Institute National Institute of Advanced Industrial Science and Technology (AIST) Tsukuba Ibaraki Japan

**Keywords:** congruency, simultaneity judgment, synchrony perception, temporal binding window

## Abstract

**Background:**

A greater congruency of audio and video expands the temporal binding window (TBW). A similar phenomenon may occur with a combination of odor and taste, which are the main components of flavor.

**Objective:**

TBW is defined as the temporal resolution of synchrony perception. The larger the TBW, the lower the resolution. We hypothesized that the more congruent the odor and taste, the lower the temporal resolution of synchrony perception.

**Methods:**

To examine this hypothesis, 10 female participants performed simultaneity judgment (SJ) tasks under congruent (soy sauce odor with saline) and incongruent (cherry tree leaf odor with saline) conditions and evaluated the congruency with saltiness for the two odors. In the SJ tasks, participants reported whether odor and taste were presented simultaneously or successively. We assumed a Gaussian distribution for the temporal distributions of the simultaneous response rates and calculated the approximations. In addition, we computed the half width at half height (HWHH) as an index of TBW based on the coefficient of approximation for the temporal distribution of the simultaneous response rates.

**Results:**

HWHH was significantly larger under congruent condition than under incongruent condition. In addition, congruency with saltiness had a significant moderate positive correlation with HWHH.

**Conclusion:**

The larger the HWHH, the lower the temporal resolution of synchrony perception, supporting the hypothesis. This study suggests that the width of TBW depends on the cross‐modal congruency similar to the case for audio–visual SJs. However, methodological improvements, including a larger sample size and gender‐independent recruitment of participants, are essential to enhance the reliability of the findings because some of the results did not provide sufficient ESs or power.

## Graphical abstract

This study examined the effect of odor–taste congruency on the temporal binding window (i.e., temporal resolution of synchrony perception) using a simultaneity judgment task. The results demonstrated that the temporal resolution of synchrony perception decreased as the odor–taste congruency increased, suggesting that the width of the temporal binding window depends on the cross‐modal congruency.

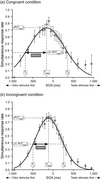

## 1 INTRODUCTION

### 1.1 Synchrony perception of multisensory information

Many events in everyday life are recorded by multiple sensory organs (King & Calvert, 2001). Humans interact with the physical world through five sensory modalities: vision, audition, taction, olfaction, and gustation. These modalities allow individuals to experience objects and events in different ways, shaping their perception, judgment, and behavior (Zhu & Mehta, 2017) (see Krishna (2012)). To accurately handle such complex sensory information, multisensory integration is necessary to process inputs from different sensory modalities and form a unified perception (i.e., a single perceptual event) (Narinesingh et al., 2017; Wahn et al., 2020).

Synchrony perception of multisensory information is a factor involved in determining whether sensory information projected from receptors of different sensory modalities to the brain via the nerves belonging to the same event (Powers et al., 2012). Temporal synchrony between cross‐modal stimuli is highly advantageous for information processing because it increases both the perceptual reliability and saliency (Keetels & Vroomen, 2012; Lim, 2016). Simultaneity judgment (SJ) or temporal order judgment (TOJ) tasks are often used to investigate synchrony perception (Yarrow et al., 2016). In SJ tasks, participants are presented with two stimuli with varying stimulus onset asynchrony (SOA), and they report whether the stimuli are presented simultaneously or consecutively; in TOJ tasks, participants report which stimuli is presented first (Harris et al., 2010).

Previous studies on temporal synchrony of multisensory information used combinations of physical stimuli such as visual, auditory, and tactile stimuli (e.g., audio–visual combinations (Li et al., 2021; Peter et al., 2019; Takeshima, 2021), audio–tactile combinations (Fujisaki & Nishida, 2009; Machulla et al., 2016; Stanley et al., 2019), and visuotactile combinations (Chen et al., 2018; Di Luca & Mahnan, 2019; Lange et al., 2018)). By contrast, the use of combinations involving chemical stimuli such as olfaction and gustation is extremely rare (Gotow & Kobayakawa, 2017, 2019, 2021). This is because advanced measurement techniques are required to strictly control gaseous odor stimuli and liquid taste stimuli (Gotow & Kobayakawa, 2014).

### 1.2 Temporal window of multisensory binding

An important aspect of the sensory integration process is the temporal binding window (TBW), which is the time range within which separate sensory inputs are likely to be integrated into a single perceptual event (Narinesingh et al., 2017). TBW has been defined as the temporal resolution of synchrony perception (Fujisaki & Nishida, 2009; Gotow & Kobayakawa, 2017, 2021), the precision of temporal judgment (Basharat et al., 2019; Yarrow et al., 2016), or the sensitivity to temporal asynchrony (Reguly et al., 2021; Tagini et al., 2020). In addition, the width of TBW can be quantified based on perceptual responses of participants to various SOAs (Narinesingh et al., 2017). For example, SJ tasks use an index calculated based on the temporal distribution of the simultaneous response rate (usually a bell‐shaped Gaussian curve). Indexes include: (1) the interval between the two SOA values corresponding to a 75% simultaneous response rate (Marsicano et al., 2022; Venskus et al., 2021; Zerr et al., 2019); (2) the interval between the SOA value corresponding to the point of subjective simultaneity and the SOA value corresponding to a 75% simultaneous response rate (just noticeable difference, JND) (Christie et al., 2019; Li et al., 2021); (3) half of the interval between the two SOA values corresponding to a 50% simultaneous response rate (*δ*) (Chen et al., 2018, 2021); (4) the standard deviation of the distribution (SD or *σ*) (Yarrow et al., 2016; Zampini et al., 2005); (5) the interval between the two SOA values corresponding to 50% of the maximum rate (full width at half height, FWHH) (Alm & Behne, 2014; Roseboom & Arnold, 2011; Roseboom et al., 2009); and (6) half of the interval between the two SOA values corresponding to 50% of the maximum rate (half width at half height, HWHH) (Fujisaki & Nishida, 2009). The larger these values, the larger the TBW, resulting in a lower temporal resolution of synchrony perception.

The width of TBW depends on a variety of factors, including combinations of cross‐modal stimuli (Fujisaki & Nishida, 2009; Gotow & Kobayakawa, 2017), presented stimuli (Stevenson & Wallace, 2013; Vatakis & Spence, 2006), tasks (van Eijk et al., 2008), the range of SOAs (Gotow & Kobayakawa, 2021), the spatial proximity between presented stimuli (Zampini et al., 2005), and training or experience (Horsfall et al., 2021; Kobayakawa, 2019; Powers et al., 2009; Yarrow et al., 2016). For example, van Wassenhove and colleagues (van Wassenhove et al., 2007) investigated the influence of the presented stimuli using SJ tasks and movies in which the utterance and lip movement were congruent (audio/da/ and video/da/) or incongruent (audio/ba/ and video/ga/). The congruent movie had a larger TBW than the incongruent one. The effect of cross‐modal congruency on the width of TBW is a reproducible phenomenon observed in both SJ and TOJ tasks (Parise & Spence, 2009; Vatakis & Spence, 2007). Cross‐modal congruency refers to the association of characteristics between different sensory modalities such as semantic associations, common characteristics shared between cross‐modal stimuli, and newly acquired associations due to frequent co‐presentation (Jensen et al., 2020). Congruency is important to the overall multisensory information processing (Fondberg et al., 2018). Along with the change in the TBW, congruency directly affects the perception of multisensory objects (e.g., flavor objects) (Amsellem & Ohla, 2016).

### 1.3 Effect of odor–taste congruency on multisensory perception

Sensations occurring simultaneously in the oral cavity are connected to a single perception of flavor (Prescott, 2015). The components that determine flavor vary among researchers: Some consider taste and odor (Gisslen, 2011; Rozin, 1982; Sims & Golaszewski, 2003), others include mouthfeel (Choi, 2014; McClements, 1999; Taylor & Linforth, 1996), while others also include appearance and sound generated during mastication (Palamand, 1993). However, most researchers agree that taste and odor are components of flavor.

In the context of flavor perception, congruency is defined as the extent to which odor and taste are appropriate for a combination in a food (Schifferstein & Verlegh, 1996). For example, odor‐induced taste enhancement is a well‐documented phenomenon in congruent olfactory–gustatory combinations (Djordjevic et al., 2004; Labbe et al., 2006; Lawrence et al., 2009; Lim et al., 2014; Nasri et al., 2011; Seo et al., 2013). Schifferstein and Verlegh (1996) reported that sweetness is enhanced by odor under congruent combinations (e.g., strawberry odor with sucrose and lemon odor with sucrose) but not under incongruent combination (e.g., ham odor with sucrose). Taste‐induced odor enhancement has also been observed for congruent olfactory–gustatory combinations, but less frequently than odor‐induced taste enhancement (Fujimaru & Lim, 2013; Green et al., 2012; Linscott & Lim, 2016). Thus, flavor perception may depend on odor–taste congruency.

In the case of audio–visual SJ tasks, cross‐modal congruency expanded TBW (Parise & Spence, 2009; van Wassenhove et al., 2007; Vatakis & Spence, 2007). Therefore, we hypothesize that the more congruent the odor and taste, the lower the temporal resolution of synchrony perception. To examine this hypothesis, herein we report the results of a study in which participants performed an SJ task under congruent (soy sauce odor with saline) or incongruent (cherry tree leaf odor with saline) conditions. The participants also evaluated perceptual dimensions of each odor (soy sauce and cherry tree leaf), including congruency with saltiness.

## 2 METHODS

### 2.1 Participants

This study was conducted in accordance with the revised version of the Declaration of Helsinki. All procedures were approved by the ethical committee for ergonomic experiments of the National Institute of Advanced Industrial Science and Technology, Japan. We explained the experiments to each participant prior to participating and informed them of their right to cease participation even after their initial consent. All participants provided written informed consent. Ten female volunteers without subjective olfactory or gustatory disorders, aged 20−38 (mean age ± SD = 26.7 ± 1.7 years old), participated in the experiment.

### 2.2 Stimuli presentation

This study used the same measurement system as previous studies (Gotow & Kobayakawa, 2014, 2017, 2019, 2021) to present odor and taste stimuli. See Gotow and Kobayakawa (2021) for details of the system.

#### 2.2.1 Odor stimuli

We presented the odor of soy sauce (7.69% v/v soy sauce [Kikkoman, Chiba, Japan] diluted in deionized water) as an odor stimulus congruent with saltiness and the odor of cherry tree leaves (68.4 mM coumarin [Wako Pure Chemical Industries, Tokyo, Japan] dissolved in propylene glycol) as an odor stimulus incongruent with saltiness. Odors were presented using an odor stimulator (Olfactometer OM4; Burghart Instruments, Wedel, Germany) developed by Kobal and colleagues (Kobal, 1985; Kobal & Hummel, 1988). The measurement system consisted of a line in which odorless air flowed, a line in which odor (scented nitrogen) flowed, and a line that exhausted gases, which were not presented to the participants. By switching the odorless air and odor lines using a three‐way solenoid valve, odor was exhausted during the odorless air presentation and odorless air was exhausted during the odor presentation.

A humidifying module filled with deionized water was placed in the middle of the odorless air line. An odor module filled with either a soy sauce solution or a cherry tree leaf solution and a heating module were placed in the middle of the odor line. Odorless nitrogen was scented by passing through the odor module. Warm water (∼40°C) was circulated around each line, the pathway of the gases, to keep the gases warm.

**FIGURE 1:**
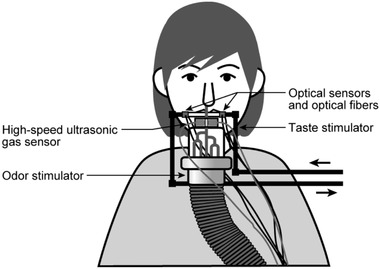
Overview of the stimulus presentation. Odor stimuli are presented by inserting a thin Teflon tube attached to the outlet of the odor stimulator into the participant's right nostril. Odor stimulus presentation is monitored in real time using a high‐speed ultrasonic gas sensor placed at the root of the Teflon tube. Taste stimuli are presented by covering a small hole drilled in the side of a Teflon tube with the center of the tip of the participant's tongue. Taste stimulus presentation is monitored in real time using optical sensors and optical fibers placed on both sides of the small hole.

A high‐speed ultrasonic gas sensor (Toda & Kobayakawa, 2008; Toda et al., 2005) and a thin Teflon tube were placed at the outlet of the odor stimulator. As shown in Figure 1, participants inserted this Teflon tube about 1 cm into the right nasal cavity. To prevent pressure and temperature changes in the nasal cavity through the tube, odorless air was continuously presented in the nasal cavity during the measurements. The odor stimulus was inserted into the flow of odorless air as a pulse. For odorless air and odor stimulus, the flow rate was set to 7.5 L per min and the presentation temperature was set to ∼36°C, which is equivalent to the temperature in the nasal cavity. Experimenters perceived the odor stimulus at an intensity of approximately "moderate: 3" on a 6‐point magnitude scale (odorless: 0, barely detectable: 1, weak: 2, moderate: 3, strong: 4, very strong: 5) (Saito, 1994).

Because the odor stimulus is a gas, there may be a slight discrepancy between the set and measured values of stimulus onset. Therefore, we conducted real‐time monitoring of stimulus presentation by converting the molecular weight of the gases (air and nitrogen) passing through the high‐speed ultrasonic gas sensor into voltage values. Based on the real‐time monitoring, the actual values of the odor stimulus onsets were obtained (see Gotow and Kobayakawa (2021) for details of the calculation method).

#### 2.2.2 Taste stimuli

We presented saline (600 mM sodium chloride dissolved in deionized water) pigmented red with food coloring as the taste stimulus, using an improved version of the taste stimulator developed by Kobayakawa and colleagues (1996, 1999). As shown in Figure 1, the participants held a Teflon tube in their mouth, which had a small hole (0.7 cm × 0.3 cm) drilled into the side, and covered the hole with the center of the tip of the tongue. Optical sensors and optical fibers were placed on both sides of the small hole. To prevent changes in pressure and temperature on the tongue, deionized water was continuously present in the tube during the measurements. The taste stimulus was inserted into a water flow as a pulse. The Teflon tube was wrapped with black tape so that the flow of taste stimulus was not visible to the participants. For deionized water and taste stimulus, the flow rates were set to 0.12 L per min and the temperature was ∼36°C, which is equivalent to the temperature on the tongue. Experimenters perceived the taste stimulus at an intensity of approximately "moderate: 3" on a 6‐point magnitude scale (Saito, 1994).

Because the taste stimulus is a liquid, there may be a slight discrepancy between the set and measured values of stimulus onset. Therefore, a blue‐green LED light was irradiated through optical fibers to the liquid flowing in the tube and received by the optical sensors. In addition, the stimulus presentation was monitored in real time by converting the difference in light transmittance between the colorless deionized water and the red‐colored taste stimuli into voltage values. The actual values of the taste stimulus onsets were obtained based on the real‐time monitoring (see Gotow and Kobayakawa (2021) for details of the calculation method).

### 2.3 Procedure

**FIGURE 2:**
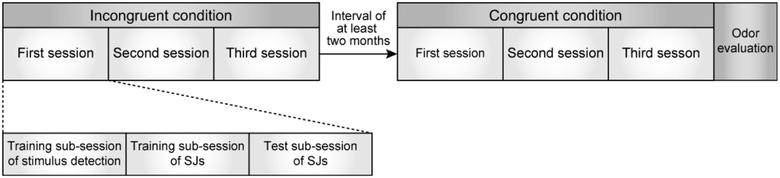
Experimental flow. Participants perform simultaneity judgment (SJ) tasks under the incongruent condition (cherry tree leaf odor with saline) followed by the congruent condition (soy sauce odor with saline). Conditions are separated by at least 2 months. Each condition consists of three sessions. Each session is divided into three sub‐sessions (i.e., training in stimulus detection, training in SJs, and testing SJs). After completing the final congruent condition session, the perceptual dimensions of the two odors (soy sauce and cherry tree leaf) are evaluated.

Figure 2 overviews the flow of the experiment.

#### 2.3.1 SJ tasks

When presenting an odor, it may be adsorbed inside the odor stimulator (specifically, inside the Teflon tube, which is the pathway for odor). To prevent mixing of odors, we disassembled the stimulator and cleaned the inside of the tube before using another odor. Due to the time required to maintain the odor stimulator, all participants performed the experiment under the incongruent condition (cherry leaf aroma with saline) followed by the congruent condition (soy sauce aroma with saline). To minimize an order effect of the conditions, the two conditions (i.e., the final incongruent condition session and the first congruent condition session) were separated by at least 2 months (65−118 days, mean ± SD = 94.20 ± 17.67 days). At the beginning of the first congruent session, we informed participants that the odor stimuli changed from those used in the previous sessions, but the taste stimuli remained the same.

Each condition consisted of three sessions. Participants performed one or two sessions on a given day. If two sessions occurred in 1 day, participants rested for about 30 min between the sessions. Each session consisted of three sub‐sessions: training in stimulus detection, training in SJs, and testing of SJs (see Gotow and Kobayakawa (2021) for details on procedures).

In each sub‐session, the green light was turned on for 7 s per trial as a fixation point and notice of stimulus presentation. Because the odor stimuli could be presented during the expiratory or inspiratory phases, the odor stimulus intensity may differ. Therefore, the participants were instructed to hold their breath when the green light was on. During each sub‐session, white noise was presented at all times.

In the training sub‐session of stimulus detection, we confirmed whether participants could detect the odor and taste stimuli used in the SJs. Due to the convenience of setting the stimulators, training to detect the odor stimulus was followed by training to detect the taste stimulus. The durations of odor and taste stimulus presentations were 400 and 500 ms, respectively, and the interval between stimulus onsets in consecutive trials was about 20 s. Participants were instructed to press the button on the response device in their self‐reported dominant hand as soon as they detected a stimulus. The detection training was complete when a response was observed within 1000 ms of stimulus onset for three consecutive trials.

The participants were also asked to identify the qualities of odor and taste during the training sub‐session of stimulus detection. Table 1 shows the responses by the participants. Participants appropriately perceived that odor stimuli differed between the congruent and incongruent conditions, and the taste stimuli were common across the two conditions. Although participants did not perform an intensity evaluation of each stimulus, we considered that they perceived each stimulus with an intensity of at least "weak: 2," which corresponds to the cognitive threshold on a six‐point magnitude scale (Saito, 1994) based on their appropriate identification of qualities of odor and taste.

**TABLE 1:** Responses of each participant to the quality of odor and taste stimuli

Participant	Soy sauce odor	Cherry leaf odor	Saline
#01	Sweet; *mitarashi dango* (skewered rice dumplings covered with a sweet soy sauce glaze)	Gentle	Salty
#02	Soy sauce	Sweet; powder; thinner	Salty
#03	*Mitarashi dango*	Asian incense	Salty
#04	Caramel; coffee; cookies; sweet and salty	Confection; fabric softener; fruity	Salty
#05	*Miso* (fermented soybean paste); almond butter	Toothpaste; sweet	Salty
#06	Sweet foods	Flowers	Salty
#07	Cinnamon	Peach; toilet air freshener; sweet	Salty
#08	Soy sauce	Sweet; sweet red bean paste	Salty
#09	Soy sauce	Cinnamon powder	Salty
#10	*Mitarashi dango*	Japanese traditional confection	Salty

In the training sub‐session of SJs, five steps of SOA were prepared. Each step was presented in the following order: 0, −800, 400, 800, and −400 ms. A negative SOA indicated that the odor stimulus was followed by the taste stimulus, and a positive SOA indicated that the taste stimulus was followed by the odor stimulus.

In the test sub‐session of SJs, we used the following 23 steps as SOAs between the odor and taste stimuli: 11 steps in which the odor stimulus was followed by the taste stimulus (odor stimulus first by 800, 600, 500, 400, 350, 300, 250, 200, 150, 100, or 50 ms; negative notations), 11 steps in which the taste stimulus was followed by the odor stimulus (taste stimulus first by 50−800 ms; positive notations), and one step in which the odor and taste stimuli were presented simultaneously (0 ms). In addition, we prepared four different stimuli sequences consisting of 92 trials. Each SOA was randomly incorporated into the sequences four times, but the same SOA was not presented in consecutive trials. For each condition and for each participant, we avoided using the same sequence repeatedly among test sub‐sessions of SJs. In both the training and test sub‐sessions of SJs, SOAs were controlled automatically by a computer.

**FIGURE 3:**
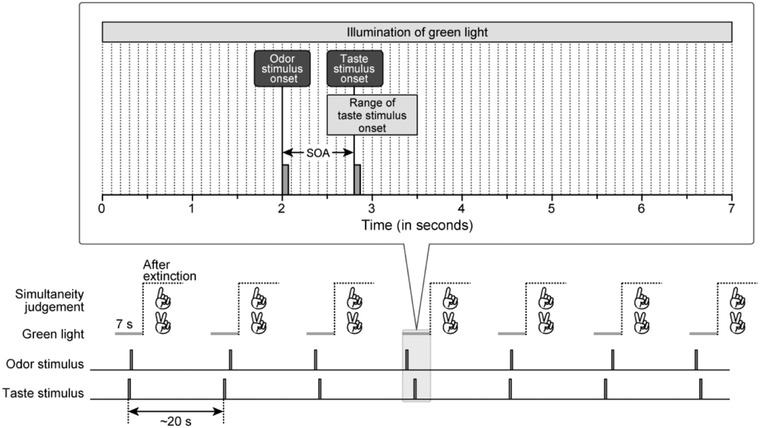
Timeline of stimulus presentation. Each test sub‐session of SJs consists of 92 trials. In each trial, a green light is turned on for 7 s as a fixation point and notice of stimulus presentation. Taste stimulus onset occurred at 3 ± 0.5 s from the illumination of illumination of the green light. Interval between taste stimulus onsets in consecutive trials is ∼20 s. Stimulus onset asynchrony (SOA) between the odor and taste stimuli is varied between trials. After the green light is turned off, participants display either “1” (simultaneous response) or “2” (consecutive response) with their fingers. In the example trial, the odor and taste stimuli are presented 2.0 and 2.8 s after illumination of the green light, respectively. In this trial, odor stimulus preceded taste stimulus by 800 ms.

As shown in Figure 3, the taste stimulus onset in each trial was adjusted to 3 ± 0.5 s after illumination of the green light. Similar to the training sub‐session of stimulus detection, the durations of odor and taste stimulus presentations were 400 and 500 ms, respectively. The interval between taste stimulus onsets in the consecutive trials was about 20 s.

After the green light was turned off, participants reported whether the odor and taste stimuli were presented simultaneously or consecutively. They were informed that a quick response was not necessary. The participants were asked to indicate “1” with their index finger if the two stimuli were perceived synchronously or “2” with their index and middle fingers if the two stimuli were perceived consecutively. If they did not perceive either stimulus or both stimuli while the green light was turned on, they did not indicate a number with their fingers. At the end of the test sub‐session, participants were asked to verbally report their perceptions of odor and taste stimuli presented in the SJ tasks. Verbal subjective responses were not included in the statistical analysis because they were not recorded as experimental data.

#### 2.3.2 Evaluation of perceptual dimensions of each odor

On the last day of the experiment (specifically, after the final congruent session), participants evaluated the perceptual dimensions of odors presented under each condition (i.e., soy sauce and cherry tree leaf odors). To present odors, we removed the odor module, which was part of the odor stimulator. Prior to the evaluation, we verbally explained the evaluation method and described it in the upper part of the questionnaire. To prevent the color of the contents filled in the odor module from affecting the evaluation, participants were asked to wear a disposable ear‐loop face mask (9.5 cm × 17 cm) as an eye mask. The experimenter brought the bottle about 10 cm from the tip of the participant's nose. Then, they smelled the odor by squeezing the side of the bottle using a sequence of three squeezes, one rest, three squeezes, one rest, and three squeezes to the sound of a digital metronome (model number DM71S; Seiko Instruments, Chiba, Japan) set at 90 beats per minute. Afterward, the participants removed the eye mask and evaluated the four perceptual dimensions using a 7‐point magnitude scale (seven long and six short vertical lines alternating at regular intervals): congruency with saltiness (not at all congruent: 0, very congruent: 6), liking (dislike very much: 0, like very much: 6), familiarity (not at all familiar: 0, very familiar: 6), and edibility (not at all edible: 0, very edible: 6). We instructed participants to mark the appropriate point on the scale, including positions between the vertical lines. The presentation order of the odors varied by participant. The presentation interval between odors was at least 3 min.

### 2.4 Analysis

#### 2.4.1 Trial selection for analysis

We calculated the actual SOA values using the real‐time monitoring of stimulus presentation (see Gotow and Kobayakawa (2021) for details of the calculation method). Trials in which a participant did not express a judgment response with their fingers as well as trials in which the actual SOA value was ≤ −1000 ms or > 1000 ms, were excluded from the analysis. Of 2760 trials (92 trials × 3 sessions × 10 participants) for each condition, we analyzed 2733 (adoption rate, 99.0%) for the congruent condition and 2713 (98.3%) for the incongruent condition.

#### 2.4.2 Temporal distribution of the simultaneous response rate and approximation

**FIGURE 4:**
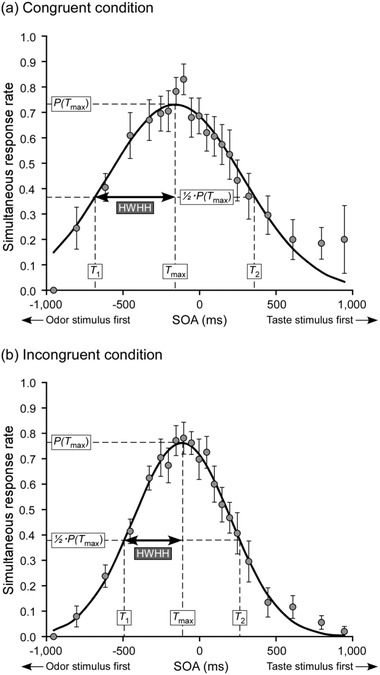
Inter‐participant average and approximate curves of the temporal distributions of simultaneous response rates for each condition. Inter‐participant average (circles) and approximate curves (solid lines) of the temporal distributions of the simultaneous response rates for each condition. Approximations are as follows: (a) $y\;=\;0.73\times \exp\{-{\left(t+0.16\right)}^{2}/\left(2\times 0.44^{2}\right)\}$ for the congruent condition and (b) $y\;=\;0.76\times \exp\{-{\left(t+0.11\right)}^{2}/\left(2\times 0.32^{2}\right)\}$ for the incongruent condition. Goodness‐of‐fit of each approximate curve to a Gaussian function is as follows: 0.98 for the congruent condition and 0.99 for the incongruent condition. In addition, *P*(*T*_max_) is the maximum value of simultaneous response rates, and 1/2 *P*(*T*_max_) is half that value. *T*_1_ and *T*_2_ are SOAs when the simultaneous response rates are 1/2∙*P*(*T*_max_). Half width at half height (HWHH) indicates the value obtained by bisecting the interval between *T*_1_ and *T*_2_. *T*_max_ is the SOA corresponding to *P*(*T*_max_). Error bars: standard error (*n* = 10)

The actual SOA values were classified into 21 time windows. We calculated the simultaneous response rates (i.e., values obtained by dividing the number of trials judged as “simultaneous” by the total number of trials) for all time windows by condition and by participant. According to previous studies (Fujisaki et al., 2004; Gotow & Kobayakawa, 2014 , 2017; Zampini et al., 2005), we assumed a Gaussian distribution for the temporal distributions of the simultaneous response rates, and calculated *a*, *b*, and *c* in $y=a\cdot \exp\{-\frac{{\left(t-b\right)}^{2}}{2\cdot c^{2}}\}$ by the least squares method. Figure 4 shows the inter‐participant average and approximate curve of the temporal distribution of the simultaneous response rates for each condition.

We defined the Pearson product–moment correlation coefficients between the actual simultaneous response rates and the theoretical values derived from the approximation as the goodness‐of‐fit of the approximation to the Gaussian function (Gotow & Kobayakawa, 2017, 2021). The goodness‐of‐fits were 0.67−0.95 (mean ± SD = 0.86 ± 0.09) for the congruent condition and 0.74−0.97 (mean ± SD = 0.92 ± 0.07) for the incongruent condition. One of the correlation coefficients calculated for the congruent condition indicated a moderate correlation (0.4 ≤ *r* < 0.7), whereas the other indicated a strong correlation (*r* ≥ 0.7) (see Dancey and Reidy (2007)). These results confirmed that the temporal distributions of the simultaneous response rates can be fitted with a Gaussian function.

#### 2.4.3 Calculation of HWHH

Consistent with previous studies on olfactory–gustatory SJs (Gotow & Kobayakawa, 2017, 2021), HWHH was used as an index of TBW. We calculated HWHH by multiplying the coefficient *c* of the approximation for the temporal distribution of the simultaneous response rates obtained by each condition and participant by $\sqrt{2ln2}$ (∼1.18). Figure 4 shows the relationship between the temporal distribution of the simultaneous response rates and HWHH. The larger the HWHH, the lower the temporal resolution of synchrony perception.

#### 2.4.4 Comparison of HWHH between the two conditions

The first analysis examined our hypothesis: The more congruent the odor and taste, the lower the temporal resolution of synchrony perception. We used the Wilcoxon signed‐rank test to evaluate whether HWHH differed between the congruent (soy sauce odor with saline) and incongruent (cherry tree leaf odor with saline) conditions.

Statistical significance is not an indicator of important or meaningful of a result (Onwuegbuzie & Leech, 2004). Therefore, to examine the practical significance of the results, we calculated effect size (ES) as a post hoc analysis. ES is an index of the degree to which the null hypothesis is false and quantifies the discrepancy between the null and alternative hypotheses (here, the difference between the two conditions) (Cohen, 1992; McCrum‐Gardner, 2010). Because each statistical test has its own ES index (Cohen, 1992), we calculated *r*, which is the ES index for the Wilcoxon signed‐rank test, according to the following formula:

$$r=z/\sqrt{n},$$

where *z* represents the statistic for Wilcoxon signed‐rank test and *n* represents the total sample size (i.e., *n* = 10 participants × 2 conditions) (Allen et al., 2019). Cohen (Cohen, 1988a) suggests that values of *r* = 0.1, 0.3, and 0.5 indicate small, medium, and large ESs, respectively.

We also calculated the power, which is the statistical sensitivity to detect an effect or relationship that actually exists (Sawyer, 1982). Power is the probability that the null hypothesis is correctly rejected when the alternative hypothesis is true, and is expressed as 1−*β*, where *β* represents a Type II error (Cohen, 1992; McCrum‐Gardner, 2010). The minimum acceptable level of power is set at 0.80 (McCrum‐Gardner, 2010). Values well below 0.80 would present too great a risk of Type II error (Cohen, 1992). In this study, we used the following parameters to obtain the power: test family = *t* tests, statistical test = means (Wilcoxon signed‐rank test [matched pairs]), type of power analysis = post hoc, tail(s) = two, parent distribution = normal, ES = Cohen's *d*_z_, significance (*α*) level = 0.05, and total sample size = 20 (Buchner et al., 2021). The ES *d*_z_ used to calculate the power was determined according to the following formula:

$$d_{z}=\left|m_{\mathrm{cong}}-m_{\mathrm{incong}}\right|/\sqrt{\left({\sigma_{\mathrm{cong}}}^{2}+{\sigma_{\mathrm{incong}}}^{2}-2\times \rho \times \sigma_{\mathrm{cong}}\times \sigma_{\mathrm{cong}}\right)},$$

where *m*_cong_ and *σ*_cong_ represent the mean and SD for the congruent condition, *m*i_ncong_ and *σ*_incong_ represent the mean and SD for the incongruent condition, respectively, and *ρ* is Spearman's rank correlation coefficient between the two conditions. Table 2a shows the mean and SD of HWHH for each condition, the correlation coefficient between the two conditions, and the ES *d*_z_.

**TABLE 2:** Statistics for the half width at half height (HWHH) and perceptual dimension

	Mean ± standard deviation					
	Congruent condition	Incongruent condition	Soy sauce odor	Cherry leaf odor	*ρ*	*d_z_*
(a)						
HWHH	0.52 ± 0.23	0.38 ± 0.12			0.71	0.81
(b)						
Congruency with saltiness			2.92 ± 1.97	0.98 ± 1.10	0.44	1.09
Liking			3.01 ± 1.00	3.00 ± 1.18	−0.43	0.01
Familiarity			4.48 ± 1.26	3.35 ± 1.13	0.10	0.70
Edibility			5.52 ± 0.67	2.96 ± 1.69	0.54	1.77

*ρ*, Spearman's rank correlation coefficient; *d_z_*, effect size.

#### 2.4.5 Calculations of the correlation coefficients between each perceptual dimension and HWHH

The second analysis also examined our hypothesis. In this analysis, Spearman's rank correlation coefficients (*ρ*) between congruency with saltiness and HWHH were calculated across conditions (i.e., using data from *n* = 10 participants × 2 conditions) to explain the variation in HWHH in terms of individual differences in congruency. The correlation strength was considered as none (*ρ* = 0), weak (0 < |*ρ*| < 0.4), moderate (0.4 ≤ |*ρ*| < 0.7), strong (0.7 ≤ |*ρ*| < 1), and perfect (|*ρ*| = 1) (Dancey & Reidy, 2007). A test of no correlation was then performed, assuming the two variables were not correlated. In addition, we calculated ES and power as post hoc analysis to examine the practical significance and statistical sensitivity of results. The correlation coefficient also serves as an ES index of relationship between variables, with *ρ* = 0.1, 0.3, and 0.5 being considered small, moderate, and large ESs, respectively (Cohen, 1992; Goss‐Sampson, 2022). We used the following parameters to obtain the power: test family = exact, statistical test = correlation [bivariate normal model], type of power analysis = post hoc, tail(s) = two, correlation coefficient of the null hypothesis = 0, ES = *ρ*, *α* level = .05, and total sample size = 20 (Buchner et al., 2021). For three perceptual dimensions (liking, familiarity, and edibility) other than congruency with saltiness, we also calculated correlation coefficients between each perceptual dimension and HWHH, performed tests of no correlation, and conducted post hoc analyses.

#### 2.4.6 Comparison of each perceptual dimension between the two odors

To confirm whether congruency with saltiness differed between soy sauce and cherry tree leaf odors, we conducted a Wilcoxon signed‐rank test. We also calculated the ES *r* and power to examine the practical significance and statistical sensitivity of results similar to the case of the HWHH comparison between the two conditions. In addition, we compared the evaluation values between the two odors and post hoc analyses for three perceptual dimensions (liking, familiarity, and edibility). Table 2b shows the mean and SD of the evaluation value for each perceptual dimension as well as the correlation coefficient between the two odors and ES *d*_z_ for each perceptual dimension.

#### 2.4.7 Calculation of the sample size based on data from a previous study

Calculating the sample size necessary to attain the desired power for a specific significance level and assumed ES before conducting a study or during the design and planning stages of a study is called a priori analysis (Cohen, 1992). We calculated the sample size using the HWHH values of olfactory–gustatory SJs under the wide‐range (|SOA| ≤ 1900 ms) and narrow‐range (|SOA| ≤ 800 ms) conditions (Gotow & Kobayakawa, 2021). Because the previous study (Gotow & Kobayakawa, 2021) had a between‐subject design, the following parameters were used: test family = *t* tests, statistical test = means (Wilcoxon–Mann–Whitney test [two groups]), type of power analysis = a priori, tail(s) = two, parent distribution = normal, ES = Cohen's *d*, significance (*α*) level = 0.05, power = 0.80, and allocation ratio (ratio of sample size for condition 2 to sample size for condition 1) = 1 (Buchner et al., 2021). By convention, the *α* level was set to .05 and the power was set to 0.80 (Cohen, 1992; Onwuegbuzie & Leech, 2004). The allocation ratio was set to 1 because when the sample size is the same for two conditions, a smaller sample size can be used to maintain the target power (Vozdolska et al., 2009). The ES *d* used to calculate the power was determined according to the following equation:

$$\begin{array}{ccc} d & = & \left|m_{\mathrm{wide}}-m_{\mathrm{narrow}}\right|\\ & & /\sqrt{\left(n_{\mathrm{wide}}\times {\sigma_{\mathrm{wide}}}^{2}+n_{\mathrm{narrow}}\times {\sigma_{\mathrm{narrow}}}^{2}\right)/\left(n_{\mathrm{wide}}+n_{\mathrm{narrow}}\right)}, \end{array}$$

where *m*_wide_, *σ*_wide_, and *n*_wide_ represent the mean and SD of HWHH and the sample size for the wide‐range condition, respectively, and *m*_narrow_, *σ*_narrow_, and *n*_narrow_ denote the mean and SD of HWHH and the sample size for the narrow‐rage condition, respectively. The ES *d* was calculated using the HWHH values for the wide‐range (mean ± SD = 0.29 ± 0.08 ms) and the narrow‐range (0.39 ± 0.11 ms) conditions, resulting in a value of 1.04.

We used the *solver* function of Excel 2010 (Microsoft Japan, Tokyo, Japan) to calculate the approximations, and *Ekuseru–Toukei* 2012 (Social Survey Research Information, Tokyo, Japan) for statistical analysis, with the α level set to .05. In addition, we used G*Power 3.1.9.7 for Windows (Buchner et al., 2020) to calculate the power and sample size (see Faul and colleagues (2009, 2007) for details on this non‐commercial program).

## 3 RESULTS

### 3.1 Comparison of HWHH between the two conditions

**FIGURE 5:**
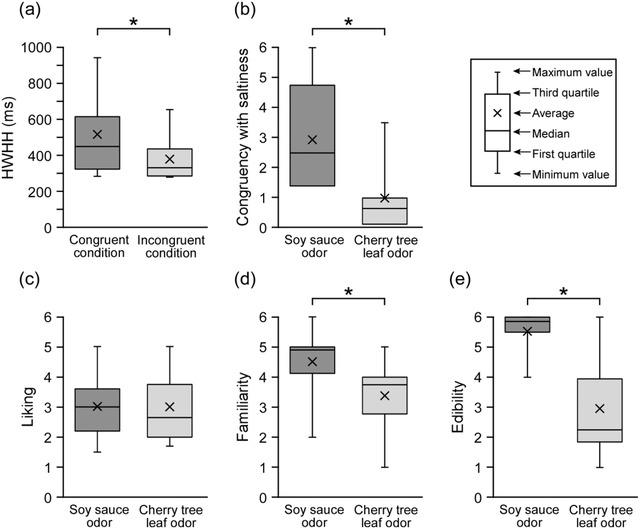
HWHH for each condition and evaluation value for the perceptual dimensions of each odor. (a) HWHH for the congruent and incongruent conditions. Wilcoxon signed‐rank test reveals a significant difference between the two conditions (*z* = 2.29, *p* < .05). (b) Congruency with saltiness, (c) liking, (d) familiarity, and (e) edibility of soy sauce and cherry tree leaf odors. Wilcoxon signed‐rank test shows significant differences between the two conditions for congruency with saltiness (*z* = 2.50, *p* < .05), familiarity (*z* = 2.43, *p* < .05), and edibility (*z* = 2.52, *p* < .05). * *p* < .05.

Figure 5a shows HWHH for the congruent and incongruent conditions. Wilcoxon signed‐rank test revealed a significant difference between the two conditions (*z* = 2.29, *p* < .05, ES *r* = 0.51, 1−*β* = 0.92). HWHH was significantly larger for the congruent condition than for the incongruent condition. The magnitude of ES was large, and the power exceeded the conventional threshold of 0.80.

### 3.2 Calculation of the correlation coefficients between each perceptual dimension and HWHH

**FIGURE 6:**
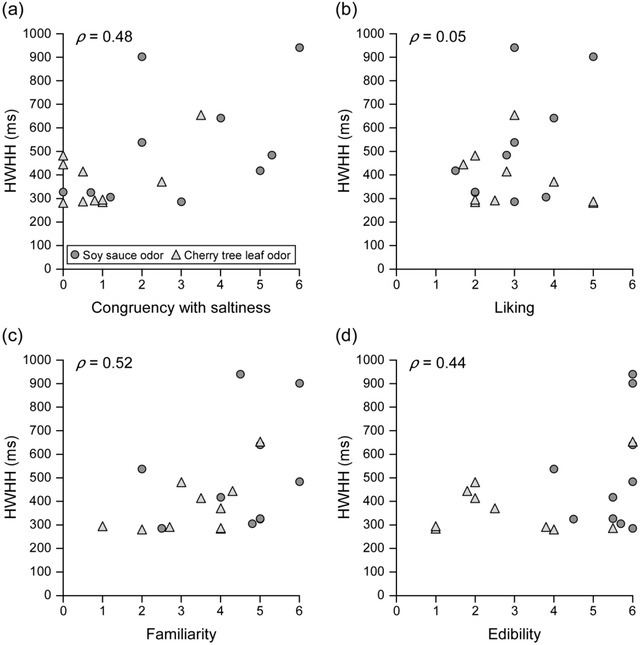
Relationship between each perceptual dimension and HWHH. Relationship between each perceptional dimension ((a) congruency with saltiness, (b) liking, (c) familiarity, and (d) edibility) and HWHH. Significant correlations occur between congruency with saltiness and HWHH (*t*(18) = 2.34, *p* < .05) and between familiarity and HWHH (*t*(18) = 2.58, *p* < .05). *ρ*: Spearman's rank correlation coefficient

Figure 6 plots the relationships between each perceptual dimension (congruency with saltiness, liking, familiarity, and edibility) and HWHH. The correlation strengths were moderate for congruency with saltiness (*ρ* = 0.48), familiarity (0.52), and edibility (0.44), but weak for liking (0.05). Tests of no correlation revealed significant correlation coefficients involving congruency with saltiness (*t*(18) = 2.34, *p* < .05, ES *ρ* = 0.48, 1−*β* = 0.60) and familiarity (*t*(18) = 2.58, *p* < .05, ES *ρ* = 0.52, 1−*β* = 0.68). The magnitude of ES for congruency with saltiness was moderate approaching large, and that for familiarity was large. However, the power was below the conventional threshold for both congruency with saltiness and familiarity.

### 3.3 Comparison of each perceptual dimension between the two odors

Figure 5b–e shows the evaluation values under the congruent and incongruent conditions for each perceptual dimension. Wilcoxon signed‐rank tests revealed significant differences between the two odors for congruency with saltiness (*z* = 2.50, *p* < .05, ES **r** = 0.56, 1−*β* = 0.99), familiarity (*z* = 2.43, *p* < .05, ES *r* = 0.54, 1−*β* = 0.82), and edibility (*z* = 2.52, *p* < .05, ES *r* = 0.56, 1−*β* = 1.00). The soy sauce odor was perceived as significantly more congruent with saltiness, familiar, and edible than the cherry tree leaf odor. For all three of these perceptual dimensions, the magnitude of ES was large, and the power exceeded the conventional threshold of 0.80.

### 3.4 Calculation of the sample size based on data from a previous study

**FIGURE 7:**
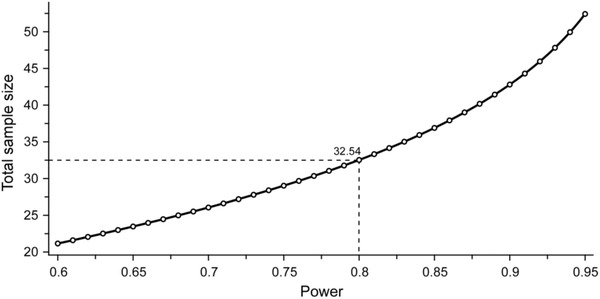
Relationship between the power and the total sample size. Using HWHH data from a previous study (Gotow & Kobayakawa, 2021), the total sample size is calculated as a function of the significance level, effect size, and power. Allocation ratio (ratio of the sample size under condition 2 to sample size under condition 1) is set to 1 (i.e., the same sample size between the two conditions). Thus, the total sample size must be an even number. Minimum even total sample size above the power threshold (0.8) is 34 participants, resulting in a sample size of 17 participants per condition.

Figure 7 plots the relationship between the power and the total sample size. The threshold for power was exceeded when the total sample size was 34 participants (i.e., 17 participants per condition).

## 4 DISCUSSION

### 4.1 Effect of odor–taste congruency on synchrony perception

This study obtained two main results in olfactory–gustatory SJs, which support our hypothesis that the more congruent the odor and taste, the lower the temporal resolution of synchrony perception. First, HWHH was significantly larger under the congruent condition than under the incongruent condition. Second, congruency with saltiness had a significant moderate positive correlation with HWHH. Therefore, this study suggests that cross‐modal congruency affects the width of TBW similar to audio–visual SJs (van Wassenhove et al., 2007). However, it should be noted that some results such as the test of no correlation did not indicate a sufficient ES or power.

Odor and taste are the two major components of flavor (Teixeira & Ferreira, 2003). An essential phenomenon in understanding flavor perception is mislocalization of odors to the mouth, which is also called oral referral (Spence, 2016). This mislocalization occurs despite the fact that human olfactory perception is initiated by activation of olfactory receptors in the nasal cavity (Kowalewski & Ray, 2020). Lim and Johnson (2011, 2012) reported a significant increase in the response of perceived odor in the oral cavity and on the tongue when an odor was presented with a congruent taste. Their results suggested that the more congruent the odor and taste are, the more likely oral referral occurs. Oral referral is observed not only when odor is presented retronasally (Ashkenazi & Marks, 2004), but also when odor is presented orthonasally (Stevenson & Mahmut, 2011). For example, Stevenson and colleagues (2011) presented odor orthonasally to participants holding a taste solution, water, or nothing in their mouth. The results revealed that participants were more likely to perceive odor in the mouth when holding a taste solution than when holding water or nothing. Similar to the studies by Stevenson and colleagues (Stevenson & Mahmut, 2011; Stevenson et al., 2011), we presented the odor stimulus orthonasally. Some participants reported that they perceived a soy sauce odor in the mouth during the measurement when performing SJ tasks under the congruent condition. However, none of the participants reported oral referral when performing SJ tasks under the incongruent condition. That is, odor and taste were more likely to be perceived as spatially proximate (i.e., both in the oral cavity) if they were congruent, even though they were presented at different spatial locations (i.e., the odor was in the nasal cavity and the taste was in the oral cavity). In audio–visual SJs, two stimuli exhibited greater TBM when presented from the same spatial location than when presented from different spatial locations (Zampini et al., 2005). Therefore, changes in the width of TBW observed in this study may also be explained in terms of the perceived spatial proximity between presented stimuli, which is closely related to odor–taste congruency.

Some researchers may consider oral referral as a type of ventriloquism effect occurring in the oral cavity (see Auvray and Spence (2008)). Ventriloquism is a classic example of multisensory information processing in which a voice appears to come from a puppet's moving mouth rather than from the actual speaker (Bruns, 2019). Ventriloquism effects are not specific to audio–visual combinations. They are also observed in audio–tactile combinations (Bruns & Röder, 2010; Caclin et al., 2002) and visuo–tactile combinations (Keetels & Vroomen, 2008; Pavani et al., 2000), where the two stimuli are presented from spatially different locations. Besides the similarity of illusion that one stimulus causes the observer to misperceive localization of another stimulus, there seems to be an important difference between oral referral and the ventriloquism effect. That is, oral referral contains the phenomenon of misperception of stimulated sensory modality (Spence, 2016), namely, the perceptual confusion between odor and taste (Rozin, 1982). In speech perception, audio (or video) presentation does not cause misperception of visual (or auditory) stimulation. However, the more congruent the odor and taste are in flavor perception, the more likely these two senses are perceptually confused (Lim et al., 2014).

The congruency with saltiness (2.92 in the range of 0−6) for the soy sauce odor used under the congruent condition was not as large as initially expected. It is possible that odor and taste may not have been treated as a “synthetic whole” (Prescott, 2015), but rather as barely perceived separately in the oral cavity. If a more congruent olfactory–gustatory combination was used, we expect the two stimuli to be perceived in the oral cavity as a complete “synthetic whole,” which is difficult to separate into different senses when judged as presented simultaneously. To test this hypothesis, it is necessary to obtain detailed subjective reports from the participants regarding their perceptions while performing SJs. In addition, if this hypothesis is correct, the temporal resolution of synchrony perception may be further reduced. Characterizing flavor objects in terms of temporal processing is a worthy topic for future research.

### 4.2 Current limitations and future directions

The durations of odor and taste stimuli in this study were very short, only a few hundred milliseconds. In addition, the taste stimulus was presented to a very narrow area on the tip of the tongue. Therefore, we considered that a high olfactory and gustatory sensitivity were required to detect and identify the stimulus. Women tend to have higher olfactory abilities than men (Sorokowski et al., 2019), and this is more pronounced among young and middle‐aged adults aged 18−50 (Wang et al., 2019). While some studies did not report gender differences in gustatory ability (Abeywickrema & Navaratne, 2018; Wang et al., 2020), others reported that women have a superior gustatory ability to men (Huang et al., 2022; Landis et al., 2009; Lim et al., 2022; Michon et al., 2009). Based on these findings, this study only included female participants. In future studies, male participants should be recruited to generalize the results. This will require methodological improvements to account for gender differences in olfactory and gustatory abilities such as preparing stimuli at different concentrations so that a given stimulus is perceived with an equivalent intensity among the participants.

All participants performed SJs in a fixed order: the incongruent condition followed by the congruent one. Therefore, the incongruent condition may serve as training for the subsequent congruent condition. In audio–visual SJs, the width of TBW decreased with training and experience (Horsfall et al., 2021; Powers et al., 2009; Yarrow et al., 2016). Contrary to audio–visual SJs, TBW expanded with task repetition in olfactory–gustatory SJs (Kobayakawa, 2019). The finding seems to imply the occurrence of a training (or learning) effect in this study. However, because the same olfactory–gustatory combination was used throughout the experiment in the previous study (Kobayakawa, 2019), it is unclear whether a training effect is maintained when different odor–taste combinations are used for each condition, which is the case in this study. Nevertheless, we cannot exclude the possibility that an order effect may have occurred. It is essential to counterbalance the order in which conditions are presented.

Odor quality is characterized by multiple perceptual dimensions (Chrea et al., 2004; Delplanque et al., 2008; Licon et al., 2018). Although this study evaluated four perceptual dimensions (congruency with saltiness, liking, familiarity, and edibility), there are other major perceptual dimensions, including intensity, pleasantness, and congruency with basic tastes other than saltiness. We should have asked participants for an intensity evaluation because intensity is a fundamental perceptual dimension in all sensory systems (Sirotin et al., 2015). In addition, some of the perceptual dimensions other than the congruency with saltiness also showed significant differences between soy sauce and cherry tree leaf odors, as well as significant correlations with HWHH. To draw a definite conclusion that the width of TBW depends on odor–taste congruency, it is necessary to make the perceptual dimensions besides congruency as equal as possible between odors.

As mentioned above, the congruent condition indicated that oral referral may have occurred in some participants. However, we did not explicitly ask the location where participants perceived the odor during the SJ tasks. Lim and Johnson (2011, 2012), who examined oral referral, used a sagittal line drawing of the human head with body parts and their names. Participants responded by selecting the location where they perceived odor from several alternatives (e.g., nose, oral cavity, tongue, and throat). To explain the width of TBW in terms of spatial proximity in the perception of odor and taste stimuli, we must verify the occurrence of oral referral based on such explicit responses.

The four parameters of statistical inference (sample size, *α* level, ES, and power) are related such that any one is a function of the other three, meaning that when any three are fixed, the fourth can be determined (Cohen, 1988b). Based on these relationships, we calculated the sample size using data from a previous study (Gotow & Kobayakawa, 2021) and estimated that at least 17 participants are necessary for a within‐subjects design of olfactory–gustatory SJs. The sample size in this study was 10 participants, which is below the required threshold. In addition, the smaller the sample size, the lower the power when a certain *α* level is set (McCrum‐Gardner, 2010), and the lower the power, the greater the likelihood that a statistically significant result at a given *α* level is a false positive (Christley, 2010). A larger sample size is needed to obtain more definitive evidence because some of the results did not provide sufficient ESs or power.

## 5 CONCLUSIONS

The more congruent the odor and taste, the lower the temporal resolution of synchrony perception. Similar to audio–visual SJs (van Wassenhove et al., 2007), this study suggests that the width of TBW depends on cross‐modal congruency. For the olfactory–gustatory combination, the width of TBW may reflect the robustness of the flavor object. However, methodological improvements are essential to increase the reliability of results due to insufficient effect sizes and power, as well as experimental procedural concerns.

## AUTHOR CONTRIBUTIONS

Naomi Gotow and Tatsu Kobayakawa designed the study. Tatsu Kobayakawa constructed the measurement system. Naomi Gotow and Tatsu Kobayakawa collected the data, analyzed the data, and wrote the manuscript. All authors reviewed the manuscript.

## CONFLICT OF INTEREST

The authors declare no competing interests.

### PEER REVIEW

The peer review history for this article is available at https://publons.com/publon/10.1002/brb3.2821

## Data Availability

The data that support the findings of this study are available from the corresponding author upon reasonable request.
